# Antibiotic Susceptibility, Virulence Pattern, and Typing of *Staphylococcus aureus* Strains Isolated From Variety of Infections in India

**DOI:** 10.3389/fmicb.2019.02763

**Published:** 2019-12-04

**Authors:** Shifu Aggarwal, Smrutiti Jena, Sasmita Panda, Savitri Sharma, Benu Dhawan, Gopal Nath, N. P. Singh, Kinshuk Chandra Nayak, Durg Vijai Singh

**Affiliations:** ^1^Infectious Disease Biology, Institute of Life Sciences, Bhubaneswar, India; ^2^Jhaveri Microbiology Centre, LV Prasad Eye Institute, Brien Holden Eye Research Centre, Kallam Anji Reddy Campus, Hyderabad, India; ^3^Department of Microbiology, All India Institute of Medical Sciences, New Delhi, India; ^4^Department of Microbiology, Institute of Medical Sciences, Banaras Hindu University, Varanasi, India; ^5^Department of Microbiology, Faculty of Medical Sciences, University of Delhi, New Delhi, India; ^6^Institute of Life Sciences, Bhubaneswar, India; ^7^Department of Biotechnology, Central University of South Bihar, Gaya, India

**Keywords:** antibiotic susceptibility, virulence, MLST, *spa*-typing, PFGE, biofilm, *Staphylococcus aureus*

## Abstract

*Staphylococcus aureus* is one of the major causes of nosocomial infections. This organism produces powerful toxins and cause superficial lesions, systemic infections, and several toxemic syndromes. A total of 109 *S. aureus* strains isolated from a variety of infections like ocular diseases, wound infection, and sputum were included in the study. Minimum inhibitory concentration (MIC) was determined against 8 antimicrobials. PCR determined the presence of 16S rRNA, *nuc*, *mecA*, czrC, *qacA/B*, *pvl*, and toxin genes in *S. aureus* isolates. Pulse-field gel electrophoresis (PFGE), multi-locus sequence typing (MLST), SCC*mec*, *spa-*, and *agr*-typing and serotyping determined the diversity among them. All isolates of *S. aureus* were resistant to two or more than two antibiotics and generated 32 resistance patterns. These isolates were positive for 16S rRNA and *S. aureus*-specific *nuc* gene, but showed variable results for *mecA*, *czrC*, and qacA/B and *pvl* genes. Of the 32 methicillin-resistant *S. aureus* (MRSA), 13 strains carried SCC*mec* type V, seven type IV, two type III, and nine carried unreported type UT6. Of the 109 strains, 98.2% were positive for *hlg*, 94.5% for *hla*, 86.2% for *sei*, 73.3% for *efb*, 70.6% for *cna*, 30.2% for *sea*, and 12.8% for *sec* genes. Serotypes VII and VI were prevalent among *S. aureus* strains. PFGE analysis grouped the 109 strains into 77 clusters. MLST classified the strains into 33 sequence types (ST) and eight clonal complexes (CCs) of which 12 were singletons, and two belong to new allelic profiles. Isolates showed 46 *spa*-types that included two new spa-types designated as t14911 and t14912. MRSA and methicillin-susceptible *S. aureus* (MSSA) isolates were diverse in terms of antibiotic resistance pattern, toxin genotypes, SCC*mec* types, serotypes and PFGE, MLST, and spa-types. However, few isolates from eye infection and wound infection belong to CC239, ST239, and *spa*-type t037/t657. The study thus suggests that *S. aureus* strains are multidrug resistant, virulent, and diverse irrespective of sources and place of isolation. These findings necessitate the continuous surveillance of multidrug-resistant and virulent *S. aureus* and monitoring of the transmission of infection.

## Introduction

*Staphylococcus aureus* commensal to human skin and mucous membranes could cause nosocomial (Lindsay and Holden, 2004) and systemic infections (Jarraud et al., 2002). The isolation of methicillin-resistant *S. aureus* (MRSA) from ocular infections varies from 3 to 30% in a hospital in India and other countries (Shanmuganathan et al., 2005; Freidlin et al., 2007). MRSA strains belonging to ST5, ST72, and ST88 and isolated from severe eye infections in India were resistant to all antibiotics except tetracycline, chloramphenicol, and cefazolin (Nadig et al., 2012). Godebo et al. (2013) showed that 94.5% of *S. aureus* isolated from wound infection were resistant to penicillin, 91.8% to ampicillin, and 76.7% to oxacillin.

Several studies have shown the presence of toxin genes among MRSA. The presence of the *sea* gene in MRSA varies from country to country (Mehrotra et al., 2000; Kim et al., 2006; Wang et al., 2013). However, *hla* gene was present in all isolates (Shukla et al., 2010). MRSA isolated from conjunctivitis in Nigeria belonging to ST88 and SCC*mec* type IV were positive for *pvl* gene (Ghebremedhin et al., 2009). However, *pvl* gene positive methicillin-susceptible *S. aureus* (MSSA) strains belonged to ST30 (D’Souza et al., 2010). *S. aureus* carrying the *pvl* gene and belonging to ST239, ST5, and ST88 was reported from a teaching hospital in China (Liu et al., 2009). MSSA belonging to ST121 and *spa*-type 287 isolated from community-acquired pneumonia in young patients carried the virulence genes (*cna* and *bbp*) and *pvl* (Baranovich et al., 2010). The role of virulence genes in *S. aureus* pathogenesis may vary from one infection type to another type of infections. Dhawan et al. (2015) reported the isolation of SCC*mec* type IV and V clones of MRSA in an Indian hospital. Several other workers also showed a decrease in SCC*mec* III MRSA isolation but increased SCC*mec* IV and V MRSA isolation (Hsu et al., 2005; D’Souza et al., 2010). Multidrug-resistant isolates belonging to ST239 and SCC*mec* type III were slowly replaced by multidrug-susceptible ST22 (SCC*mec* type IV) and ST772 (SCC*mec* type V) in hospitals (D’Souza et al., 2010).

Several molecular biology techniques like multi-locus sequence typing (MLST), pulse-field gel electrophoresis (PFGE), SCC*mec* typing, and *spa*-typing have been used to study epidemiology and clonal diversity of *S. aureus* (Maslow et al., 1993; Norazah et al., 2001; Ghaznavi-Rad et al., 2011). However, not a single technique alone could discriminate the bacteria because of differences in the degree of typeability, reproducibility, and discriminatory power (Tenover et al., 1994). Overall analysis of different typing techniques can provide information on diversity of the isolates that can be useful for outbreak investigations. In India, *S. aureus* is rated as one of the major pathogen causing a variety of infections and showing resistance to several antibiotics; however, not much information is available on their antibiotic susceptibility, virulence profile, and genomic diversity. In this study, our aim was to determine the antibiotic susceptibility pattern, virulence profiles, and genomic diversity among MRSA and MSSA isolated from patients with a variety of infections, including ocular diseases and collected from different parts of India from 2007 to 2015. Genetic, serotype, and phenotypic data were used to determine whether isolates from a variety of infections had similar characteristics.

## Materials and Methods

### Bacterial Strains

A total of 109 *S. aureus* strains isolated from patients visited/admitted to hospitals with infections in different part of India between July 2007 and November 2015 were included in the study. These isolates were from LV Prasad Eye Institute, Bhubaneswar (*n* = 54), comprised of microbial keratitis (*n* = 18), eyelid abscess (*n* = 8), endophthalmitis (*n* = 5), Steven Johnson syndrome with bacterial keratitis (*n* = 9), suture-related infections (*n* = 3), and other ocular infection (*n* = 5); LV Prasad Eye Institute, Hyderabad (*n* = 10) comprised of cornea scrapping (*n* = 5), pus from eye (*n* = 4), and suture-related infections (*n* = 1); Institute of Medical Sciences, Banaras Hindu University, Varanasi (*n* = 21) comprised of wound infection (*n* = 16) and unknown sources (*n* = 5); All India Institute of Medical Sciences, New Delhi (wound infection *n* = 10); and University College of Medical Sciences, Delhi (wound infection *n* = 9). Also, five isolates were from the conjunctiva of the asymptomatic healthy volunteers LV Prasad Eye Institute, Bhubaneswar. We conducted the study following the guidelines mentioned in the Declaration of Helsinki. We identified all the 109 isolates by using biochemical tests including Gram staining, catalase production, fermentation of glucose and mannitol, and ID32 STAPH strips using ATB^TM^ NEW v.1.0.0 software on an ATB^TM^ reader (bioMerieux, France) (Panda et al., 2014). The amplification of the *S. aureus nuc* gene confirmed the identity of isolates (Hirotaki et al., 2011). We used *S. aureus* ATCC 25293 and *S. aureus* ATCC 29213 as quality control strains for antibiotic susceptibility testing, and *S. aureus* ATCC 25923 and ATCC 43300 as a reference for serotyping, PFGE, MLST, and *spa*-typing.

### Coagulase Gene Typing

Coagulation-inhibition test with coagulase type I–VIII-specific antisera (staphylococcal coagulase antiserum kit; Denka Seiken, Inc., Tokyo, Japan) was conducted to determine the coagulase type of *S. aureus* following the manufacturer’s instructions (Goh et al., 1992). Briefly, a single colony for each test strain was suspended in BHI broth (Becton Dickinson Co.) and incubated at 37°C for overnight. Then centrifuged the culture and 0.1 ml of the supernatant used as test antigen. Distributed an aliquot (0.1 ml) of the test antigen into ten tubes followed by addition of 0.1-ml aliquots of anticoagulase types I–VIII sera to first eight tubes, except 9th and 10th tubes which were used as positive and negative controls and incubated at 37°C for 1 h. After that, 0.2 ml of diluted rabbit plasma was added to each tube and incubated at 37°C for 1 h. Visual inspection judged the coagulation of plasma after 2, 4, 24, and 48 h and accordingly, strains were typed based on results obtained with staphylocoagulase reaction showing coagulation inhibition.

### Minimum Inhibitory Concentration (MIC) Determination

Minimum inhibitory concentrations (MICs) of oxacillin, chloramphenicol, vancomycin, tetracycline, gentamicin, erythromycin, clindamycin, and trimethoprim were determined by broth microdilution methodology as recommended by the CLSI breakpoints. The 96-well plates were incubated at 37°C and were read for turbidity after 24 h.

### Polymerase Chain Reaction (PCR) Assays

The presence of genes encoding for methicillin resistance (*mecA*), the nuclease (*nuc*), Panton-Valentine leukocidin (*pvl*), cadmium resistance (*czrC*), and quaternary ammonium resistance (*qacA/B*) was determined by hexaplex PCR (Panda et al., 2014). PCR identified the presence of *msrA, ermA, ermC* (erythromycin resistance), *tetK* (tetracycline resistance) genes (Duran et al., 2012). Also, PCR determined the presence of gene encoding for resistance to aminoglycosides [*aac (6’)/aph (2)*, *aph (3’-III)*] by the method described earlier (Schmitz et al., 1999). The presence of *catpC221*, *catpC223*, and *catpC194* (chloramphenicol resistance) was determined by PCR as described by Argudín et al. (2011). The *mphC* (clindamycin resistance) gene was detected by PCR method described earlier (Panda et al., 2016).

### SCC*mec* Typing

Two PCRs, MPCR1 and MPCR2 were used to detect the presence of *mec* complex, *ccr* complex, and SCC*mec* type among *S. aureus* (Kondo et al., 2007).

### Virulence Gene Profile and Accessory Gene Regulator (*Agr*) Typing

PCR determined the presence of Staphylococcal enterotoxin (SE) genes encoding for *seA*, *seC*, and *seI* (Monday and Bohach, 1999; Jarraud et al., 2002). Also, the presence of hemolysin genes, *hl*A and *hlG*, was determined by PCR (Mitchell et al., 2010; Paniagua-Contreras et al., 2012). PCR was used to detect the presence of collagen adhesion (*cna*) and extracellular fibrinogen binding protein (*efb*) among *S. aureus* strains (Zecconi et al., 2006). The presence of intracellular adhesion genes (*icaA*, *icaD*) was determined by PCR as described by Arciola et al. (2001). PCR amplification was carried out to determine the presence of *agr* alleles using group-specific primers as described by Gilot et al. (2002).

### Pulsed-Field Gel Electrophoresis (PFGE)

Pulsed-field gel electrophoresis of *S. aureus* genomic DNA digested with *Sma*I (NEB) was carried out by the protocol described for *S. aureus* by Centre for Disease Control and Prevention. The dendrogram of similarity showing the clustering of the isolates according to banding patterns was generated with Bionumerics software, version 7.1 (Applied Maths, Belgium) using the Dice index and the un-weighted pair group method with arithmetic average (UPGMA) with 0.5% optimization and 1% position tolerance. Isolates showing similarity coefficient of up to 80% were considered belonging to similar pulsotype (Van Belkum et al., 2007).

### Multi-Locus Sequence Typing (MLST)

The internal fragments of seven housekeeping genes, viz., *arcC*, *gmk*, *aroE*, *glpF*, *pta*, *tpi*, and *yqil* were amplified by PCR method described earlier (Enright and Spratt, 1999). The amplified products were purified (ExoSAP; Affymetrix, Cleveland, OH, United States) and both strands sequenced using an ABI sequencer model 3500 (Life Technologies, Marsiling, Singapore) at the sequencing facility of the Institute of Life Sciences (Bhubaneswar, India). The nucleotide sequences were aligned using Mega 5.2 software. After manually comparing with reported alleles, STs were assigned accordingly. Sequencing was performed in biological duplicates to confirm the presence of novel alleles.

The advanced cluster analysis was performed to define the clonal complexes (CCs) by using Bionumerics software, version 7.1 (Applied Maths, Belgium). A minimum spanning tree (MST) was constructed using the MLST data and partitions were created to form clusters. The similarity in at least six alleles grouped isolates of *S. aureus* in one CC. The central ST of each separation was used to designate a CC.

### *Spa-*Typing

PCR amplified the polymorphic X region of *Staphylococcus* protein A (*spa*) gene following the conditions mentioned earlier (Nelson et al., 2007). Amplified products were purified, and both strands were sequenced using an ABI sequencer model 3500 (Life Technologies, Marsiling, Singapore) at the sequencing facility of the Institute of Life Sciences (Bhubaneswar, India). The nucleotide sequences were aligned using Mega 5.2 software. Repeat succession in the polymorphic X-region assigned the *spa*-types, and accordingly the MST was generated using Bionumerics 7 software (Applied Maths, Belgium) using gap creation cost 250%, gap extension cost 50%, duplicate production cost 25%, duplicate expansion cost 25%, and maximum duplication three repeats.

### Statistical Analysis

We performed principal coordinates analysis (PCoA) and discriminant analysis (DA) using PAST program v2.17 for the antibiotic resistance genes and virulence genes in MRSA and MSSA isolates with regard to sources of isolation (Hammer et al., 2001). We carried out the DA using default values to confirm the hypothesis of whether MRSA and MSSA isolates are different.

## Results

### Hexaplex PCR

All the isolates of *S. aureus* were positive for 16S rRNA and *S. aureus*-specific *nuc* genes. Hexaplex PCR discriminates between MSSA and MRSA isolates. Thirty-one of 109 (29.4%) methicillin-resistant strains were positive for the *mecA* gene, and 77 (70.6%) methicillin sensitive isolates were negative for the *mecA* gene. One of the methicillin-resistant strains of *S. aureus* was negative for the *mecA* gene. Among 109 isolates, 43 (39.4%) isolates comprising 23 of the 77 (29.9%) MSSA and 20 of the 31 (64.5%) MRSA isolates were positive for *pvl* gene. Of the 31 MRSA isolates, two (6.5%) strains were positive for the *czrC* gene and four (12.9%) isolates were positive for *qacA/B* gene, and remaining isolates were negative for both *czrC* and *qacA/B* genes (data not shown).

### Coagulase Serotyping

Serotyping classified *S. aureus* isolates into I–VIII serotypes by using coagulase typing scheme. Twelve of the 109 (11%) strains belong to serotype I, 11 (10%) to serotype II, nine (8%) to serotype III, 14 (12.8%) to serotype IV, 12 (11%) to serotype V, 19 (17.4%) to serotype VI, 20 (18.3%) to serotype VII, and 12 (11%) to serotype VIII, respectively. Nine of 31 (29%) MRSA belong to serotype VI and 17 of 78 (21.8%) and MSSA isolates belong to serotype VII (Table 1). Nine of the 24 (37.5%) isolates from wound infection belong to serotype VI and 16 of 64 (25%) isolates from eye infection belonged to serotype VII.

**TABLE 1:** Antibiotic resistance patterns and presence of antibiotic resistance genes in *Staphylococcus aureus* isolates from different parts of India.

**Phenotypic antibiotic resistance pattern**	**Number of isolates showing presence of gene(s) encoding for**																	
	
	**MRSA**	**MSSA**	***mecA***	***aac(6’)/ aph(2)***	***aph (3’III)***	***msrA***	***ermA***	***ermC***	***mphC***	***tetK***	***tetL***	***tetM***	***cat::pC221***	***cat::pC223***	***cat::pC194***	***dfrA***	***dfrB***	***dfrG***
OX, CHL, TET, GEN, ERY, CL, TMP	10	0	10	**10**	**10**	–	**10**	**10**	**10**	**10**	**10**	**10**	**10 (3)**	–	**10**	**10**	**10**	**10**
OX, CHL, ERY, TMP	0	1	–	1	–	**1**	–	**1 (1)**	**1**	–	–	1	**1 (1)**	–	**1**	–	–	**1**
CHL, ERY, TMP	0	11	–	11	11	–	**11 (3)**	**11**	**11**	–	11	11	**11 (3)**	**11**	–	**11**	**11**	**11**
CHL, TMP	0	6	–	6	–	–	–	+	–	–	6	6	**6 (2)**	–	**6**	–	**6**	**6**
OX, CHL, TET, ERY, TMP	3	0	3	3	3	**3**	**3(1)**	**3**	**3**	**3**	**3**	**3**	**3 (1)**	–	–	–	**3**	**3**
OX, CHL, GEN, ERY, TMP	11	0	11	**11**	**11**	**11**	**11 (5)**	**11**	**11**	–	**11**	**11**	**11 (2)**	**11**	**11**	–	**11**	**11**
OX, CHL, TET, GEN, ERY, TMP	5	0	5	**5 (1)**	**5**	**5**	–	**5**	**5**	**5**	**5**	**5**	**5 (1)**	**5**	–	**5**	**5**	**5**
ERY, CL, TMP	0	1	–	–	1	**1**	**1**	**1**	–	–	–	–	–	–	–	–	**1**	–
CHL, ERY, CL, TMP	0	2	–	2	2	–	–	**2(1)**	**2(1)**	–	–	2	**2 (1)**	**2**	–	**2**	**2**	**2**
CHL, ERY, **CL**	0	13	–	13	13	**13**	**13(9)**	**13**	**13(1)**	13	13	13	**13 (3)**	**13**	–	13	13	13
CHL	0	3	–	–	–	3	–	3	–	3	3	3	**3 (1)**	**3**	–	3	3	3
CHL, TET, GEN, ERY, **CL**, TMP	0	3	–	–	**3**	**3**	–	**3**	–(2)	**3**	–	**33**		**3**	–	**3**	**3**	**3**
CHL, TET, GEN, TMP	0	1	–	**1**	**1**	–	–	1	–	**1**	–	–	**1 (1)**	–	–	–	–	–
CHL, GEN, ERY, CL, TMP	0	2	–	**2 (1)**	**2**	–	–	**2(2)**	**2(2)**	2	2	2	**2**	**2**	–	–	**2**	**2**
CHL, GEN, ERY, **CL**, TMP	0	7	–	**7 (2)**	**7**	**7(3)**	–	–	−(3)	7	7	7	**7**	**7**	**7**	**7**	**7**	**7**
CHL, TET, GEN, ERY, CL, TMP	0	1	–	–	**1**	–	–	–	**1(1)**	**1**	–	–	**1**	**1(1)**	–	**1**	**1**	**1**
CHL, GEN, ERY	0	3	–	**3**	**3**	**3 (1)**	–	**3 (1)**	–	–	3	3	**3**	**3**	–	3	3	3
CHL, TET, GEN, ERY	0	3	–	**3 (2)**	**3**	**3**	–	**3(1)**	–	**3**	**3**	**3**	**3 (1)**	**3**	–	3	3	3
GEN, ERY, CL, TMP	0	1	–	**1 (1)**	**1**	–	–	**1**	**1**	1	–	1	1	1	–	**1**	**1**	**1**
GEN, ERY	0	2	–	–	**2(1)**	**2**	–	**2(1)**	–	–	2	2	2	2	–	2	2	2
ERY	0	4	–	4	4	**4**	–	**4(2)**	–	4	–	4	4	4	–	4	4	4
ERY, TMP	0	3	–	3	3	**3**	–	**3(3)**	–	–	3	3	3	3	–	**3**	**3**	**3**
OX, CHL, GEN, **ERY**	1	0	1	–	**1**	–	–	– (1)	–	1	–	1	**1**	**1**	–	1	1	1
CHL, TET, **ERY**, TMP	0	2	–	2	2	–	–	2 (1)	–	**2**	**2**	**2**	**2 (1)**	**2**	–	**2**	**2**	**2**
CHL, ERY, CL	0	1	–	–	1	**1**	–	**1(1)**	**1**	–	1	1	**1**	**1**	–	1	1	1
GEN, ERY, TMP, **CHL**	0	1	–	**1**	**1**	**1**	–	**1**	–	–	–	1	1 (1)	–	–	–	**1**	**1**
CHL, CL, TMP	0	2	–	2	2	2	–	2	2	–	2	2	**2**	**2**	**2**	–	**2**	**2**
TET, TMP	0	1	–	1	1	1	–	1	–	**1**	**1**	**1**	1	1	–	–	**11**	
ERY, CL, **CHL**	0	1	–	1	1	**1**	–	**1**	**1(1)**	1	1	1	1 (1)	1	–	1	1	1
TET, GEN, **CL**, ERY	0	2	–	–	**2**	**2**	–	**2**	−(1)	–	–	**2**	2	2	–	2	2	2
OX, CHL, ERY, **CL**, TMP	1	0	1	1	1	**1**	–	**1**	−(1)	1	1	1	**1**	**1**	–	–	**1**	**1**
CHL, TET, GEN	0	1	–	**1**	**1**	–	–	–	–	–	**1 (1)**	**1**	**1**	**1**	–	–	1	1

*MRSA: methicillin-resistant *Staphylococcus aureus*; MSSA: methicillin-susceptible *Staphylococcus aureus*, OX: oxacillin, GEN: gentamicin, ERY: erythromycin, TET: tetracycline; CL: clindamycin; CHL: chloramphenicol; TMP: trimethoprim. Isolates showing phenotypic resistance to given antibiotic(s) are shown in bold. Number in brackets indicate phenotypic sensitive isolates.*

### Antibiotic Resistance Genes

One hundred two of the 109 *S. aureus* isolates were multidrug resistant showing resistance to two or more antibiotics. All the strains were susceptible to vancomycin when tested by broth microdilution assay. Thirty-one isolates of *S. aureus* were resistant to oxacillin and carried the *mecA* gene; however, one isolate of *S. aureus* resistant to oxacillin was negative by PCR for the *mecA* gene. The remaining 77 isolates were sensitive to oxacillin and negative by PCR for the *mecA* gene (Table 1).

Ninety-five isolates of *S. aureus* resistant to chloramphenicol carried *cat: pC221* gene; however, 86 isolates carried *cat: pC223* and 37 isolates carried *cat: pC194* gene, respectively. Twenty isolates carried all the three genes tested; however, 83 isolates were positive for *cat: pC221* and *cat: pC223* and 37 isolates for *cat: pC221* and *cat: pC194* genes, respectively (Table 1). One of the isolates sensitive to chloramphenicol was negative by PCR for all three genes. In contrast, 15 strains of *S. aureus* susceptible to chloramphenicol were positive for *cat: pC221* and 14 for *cat: pC223* genes, respectively.

Twenty-nine isolates were phenotypically resistant to tetracycline of which 29 isolates were positive for *tetK*, 25 for *tetL*, and 28 for *tetM* genes. Twenty-five isolates carried all the three genes tested; however, three strains carried *tetK* and *tetM* genes and one isolate *tetL* and *tetM* genes. In contrast, 76 isolates sensitive to tetracycline were positive for the *tetM* gene, 66 for *tetL*, and 29 for *tetK* genes. Among them, 27 isolates carried all the three genes, six had *tetK* and *tetM*, and 39 strains had *tetL* and *tetM* genes, respectively. One isolate sensitive to tetracycline was negative by PCR for all three genes tested (Table 1).

A total of 54 isolates were resistant to gentamicin of which 45 isolates were positive for *aac(6’)/aph(2’)* and *aph (3’-III)* genes and nine isolates for *aph (3’-III)* gene only. In contrast, 43 gentamycin sensitive isolates showed positive results for *aac(6’)/aph(2’)* and *aph (3’-III)*, seven isolates for *aac(6’)/aph(2’)*, and two isolates for *aph (3’-III)* genes. However, 56 isolates sensitive to gentamicin were negative by PCR for *aac(6’)/aph(2’)* and *aph (3’-III)* genes (Table 1).

Of the 91 isolates of *S. aureus* showing resistance to macrolides carried erythromycin resistance genes. Twenty-eight isolates carried all the erythromycin resistance genes, namely, *msrA*, *ermA*, and *ermC.* Fifty-one isolates were positive for two genes, of which 30 isolates carried *msrA* and *ermC* genes, and 21 strains had *ermA* and *ermC* genes. Besides, 12 isolates were positive for a single gene of which five isolates carried the *ermC* gene, and seven isolates had *msrA* gene. In contrast, two of the 10 erythromycin sensitive isolates carried *msrA* and *ermC* genes, four strains possess *msrA* and *ermC* genes, and three isolates had the *ermC* gene. Of the 64 isolates carrying the *mphC* gene, 22 isolates were phenotypically resistant to clindamycin (Table 1). None of the 17 strains showing sensitivity to erythromycin carried any of the erythromycin resistance genes. One of the resistant isolate not carrying any of the erythromycin resistant genes is likely to be mediated by an as-yet-unknown mechanism.

Similarly, 74 isolates were resistant to trimethoprim of which 45 isolates were positive for *dfrA*, *dfrB*, and *dfrG* genes, 27 strains for *dfrB* and *dfrG* genes, and one isolate each for *dfrB* and *dfrG* genes, respectively. In contrast, 34 isolates sensitive to trimethoprim were also positive for *dfrA*, *dfrB*, and *dfrG* genes; however, one strain was positive for the *dfrG* gene (Table 1).

### D-Test and Macrolide Resistance

Ninety of 109 (89.9%) *S. aureus* isolates that exhibited erythromycin resistance were evaluated for MLSB resistance phenotype, namely, iMLSB, cMLSB and MSB. Seventy eight of 90 (79.5%) isolates were erythromycin-resistant but clindamycin susceptible were tested for D-test. We found 14 isolates (10 MRSA and four MSSA) showed iMLSB phenotype, and 12 (two MRSA and 10 MSSA) had MSB phenotype. Seven erythromycin-resistant isolates comprising six MRSA and one MSSA had cMLSB phenotype. The remaining 45 isolates (14 MRSA and 31 MSSA) did not show any MLSB phenotypes.

Among MRSA and MSSA showing cMLSB resistance phenotype, three of six MRSA isolates possessed the *ermA* and *ermC* genes and one each possessed *ermC* gene, *msrA, ermC*, *mphC* genes, and *ermC* and *mphC* genes. One MSSA isolate was positive for *msrA*, *ermA*, and *ermC* genes. On the hand, one of the two MRSA isolates showing MSB phenotype had *msrA*, *ermC* genes and other strain had *msrA*, *ermC*, and *mphC* genes (Table 2). Of the 12 MSSA, six isolates contained *msrA* and *ermC* genes, one isolate each contained *ermC* and *ermA* genes, respectively, two strains had *mphC* gene. The remaining isolates did not carry any of the genes tested. Of the 10 MRSA, six isolates with iMLSB phenotype had *ermC* gene. One isolate each carried *msrA*, *ermA*, and *ermC* genes, *ermA, ermC* genes, *msrA*, *ermC*, and *mphC* genes, respectively. The remaining one isolate did not possess any of the resistance genes. Of the four MSSA isolates that showed iMLSB phenotype, three strains were positive for *msrA*, *ermC* genes, and one isolate was positive for *ermC* gene (Table 2).

**TABLE 2:** Result of D-test obtained with MRSA and MSSA isolates showing presence of erythromycin resistance genes and its correlation with MLSB phenotypes among *Staphylococcus aureus*.

**Erythromycin resistance and MSB phenotypes**	**Phenotype (%)**	**Gene combinations**									
		
		***msrA***	***ermA***	***ermC***	***mphC***	***msrA, ermC***	***ermA, ermC***	***ermC, mphC***	***msrA, ermA, ermC***	***msrA, ermC, mphC***	***msrA, ermA, ermC, mphC***
**MRSA (n=32)**											
ER-S, CL-S	10 (31.25%)	0	0	0	0	3 (30%)	0	1 (10%)	1 (10%)	3 (30%)	1 (10%)
ER-R, CL-S (MSB phenotype)	2 (6.25%)	0	0	0	0	1 (50%)	0	0	0	1 (50%)	0
ER-R, CL-R (cMLSB phenotype)	6 (18.75%)	0	0	1 (16.6 %)	0	0	3 (50%)	1 (16.6%)	0	1 (16.6%)	0
ER-R, CL-D (iMLSB phenotype)	10 (31.25%)	0	0	6 (60%)	0	0	1 (10%)	0	1 (10%)	1 (10%)	0
**MSSA (n=77)**											
ER-S, CL-S	52 (67.5%)	3 (5.7%)	1 (1.9%)	16 (30.7 %)	1 (1.9%)	20 (38.4%)	0	4 (7.6%)	0	4 (7.6%)	0
ER-R, CL-S (MSB phenotype)	12 (15.5%)	0	1 (8.3%)	1 (8.3%)	2 (16.6 %)	6 (50%)	0	0	0	0	0
ER-R, CL-R (cMLSB phenotype)	1 (1.29%)	0	0	0	0	0	0	0	1 (100%)	0	0
ER-R, CL-D (iMLSB phenotype)	4 (5.19%)	0	0	1 (25%)	0	3 (75%)	0	0	0	0	0

*S: sensitive; R: resistance; ER: erythromycin; CL: clindamycin.*

Of the 109 *S. aureus* isolates tested for the presence of MLSB resistance genes, 102 isolates carried one or more *erm* genes. Three strains carried all the erythromycin resistance genes, namely, *msrA*, *ermA*, and *ermC.* Fifty-one isolates were positive for two genes, of which 46 isolates carried *msrA* and *ermC* genes, and five had *ermA* and *ermC* genes. Besides, 37 isolates were positive for a single gene of which 34 isolates carried the *ermC* gene, two isolates had *ermA* gene, and three isolates had the *msrA* gene (Table 2). In contrast, four of the 13 erythromycin-sensitive isolates carried *msrA* and *ermC* genes. One strain each had the *ermC* gene and *msrA* gene. The remaining isolates did not carry any resistance genes. Twelve of the 21 *mphC* gene-positive isolates showed phenotypic resistant to clindamycin. The remaining nine isolates were sensitive to clindamycin (Table 2). Eight erythromycin-resistant strains did not carry any of the erythromycin-resistant genes is likely to be mediated by an as-yet-unknown mechanism.

### SCC*mec* Typing

The presence of the *mec* complex and *ccr* complex classified *S. aureus* strains into different SCC*mec* types. Thirty-one MRSA isolates showed four known SCC*mec* types of which 13 (40.6%) belong to type V, nine (28.1) belong to type UT6, seven (21.9%) belong to type IV, and two (6.3%) belong to type III (Table 3). One isolate showing phenotypic resistance to methicillin but negative for *mecA* gene carried C1 type of *ccr* complex but lack *mec* complex. Of the 32 methicillin-sensitive isolates lacking the *mec* complex, 14 isolates carried *ccrA1B1*, one strain possesses *ccrA4B4*, and 17 isolates had *ccrA3B3* and *ccrA4B4* type of *ccr* complex, respectively (Table 3).

**TABLE 3:** Distribution of SCC*mec* types among *S. aureus* strains isolated from wound and ocular infection.

**Distribution of SCC*mec* types among *S. aureus* strains isolated from wound and ocular infection**						

**SCC*mec* type**	**Recombinase complex**	***mecA* complex**	**Source of infection**			**Total no. of isolates (*n* = 109)**
				
			**Wound (n = 34)**	**Ocular (*n* = 69)**	**Unknown (*n* = 6)**	
III	ccrC1, ccrAB3	Class A	2	0	0	2(1.8%)
IV	ccrAB2	Class B	7	0	0	7(6.4%)
V	ccrC1	Class C2	5	4	4	13(11.9%)
UT6	ccrC1	Class A	5	3	1	9(8.2%)
Untypable-1	ccrC1	–	1	0	0	1(0.91%)
Untypable-2	ccrAB4	–	0	1	0	1(0.91%)
Untypable-3	ccrAB1	–	0	14	0	14(12.8%)
Untypable-4	ccrAB2	–	0	1	0	1(0.91%)
Untypable-5	ccrAB3	–	0	1	0	1(0.91%)

### Toxin Gene Profiles

Of the 109 isolates, 34 (31.2%) isolates harbored *sea* gene, 14 (12.8%) isolates *sec* gene, 93 (85.3%) isolates *sei* gene, 76 (69.7%) *cna* gene, 101 (92.6%) isolates *hla* gene, 107 (98%) isolates *hlg* gene, and 84 (77%) isolates carried *efb* gene, respectively. All the isolates, except one isolate, was positive for the *hlg*, and carried multiple virulence genes (Table 4).

**TABLE 4:** Source, clonal complex, sequence-, *spa*-, SSC*mec*-, and *agr*-types and virulence profiles of *S. aureus* isolated from different parts of India.

**Source (isolate number)**	**CC/ST, *spa*-type**	***SCCmec* type**	***agr* type**	***pvl* gene**	***icaA/icaD***	**Serotypes**	**Virulence pattern**
**MRSA (*n* = 32)**							
Wound infection (2095)	239/239, t037	III	1	–	+/+	II	*sei-cna-hla-hlg-efb*
Wound infection (2103)					+/+	IV	*sea-sei-cna-hla-hlg-efb*
Wound infection (2656)	239/239,t037	UT6	1	–	+/+	IV	*sea-sei-cna-hla-hlg-efb*
Wound infection (22/248)					+/+	III	*sea-cna-hla*
Wound infection (UC650)					+/+	IV	*sea-cna-hla-hlg-efb*
Wound infection (UC858)					−/−	V	*sea-hla-hlg-efb*
Wound infection (UC1079)	239/239,t2952	UT6	1	–	+/+	I	*sea-sei-cna-hla-hlg-efb*
Wound infection (2658)	239/241,t037	UT6	1	–	+/+	IV	*sei-cna-hlg-efb*
Eye infection (P844628, N307002)	239/239, t037	UT6	1	–	+/+	IV	*sei-cna-hla-hlg*
					±		
Eye infection (P853836)	239/239, t037	UT6	1	–	±	V	*sea-cna-hla-hlg-efb*
Wound infection (2380,2452)	772/772, t657	V	None	+	+/+	VI	*sea-sec-sei-cna-hla-hlg-efb*
					+/+		
Wound infection (UC609)	772/772, t657	V	2	+	+/+	VI	*sea-sec-sei-cna-hla-hlg-efb*
Wound infection (22/252)	772/Unk, t657	V	None	+	−/−	VI	*sea-sec-sei-cna-hla-hlg-efb*
Eye infection (845)	772/772, t345	V	3	+	−/+	I	*sea-sec-sei-cna-hla-hlg-efb*
Eye infection (1295)	2884/88, t2526	V	2	+	+/+	III	*sei-hla-hlg-efb*
Eye infection (1690)	5/5, t442	V	1	–	+/+	IV	*sei-hla-hlg-efb*
Eye infection (1820)	772/772, t657	V	1	+	+/+	VII	*sea-sec-sei-cna-hla-hlg-efb*
Unknown (1189)	772/772, t657	V	2	+	+/+	VI	*sec-sei-cna-hla-hlg-efb*
Unknown (1192,1249)	772/772, t345	V	2	+	+/+	VII	*sea-sei-cna-hla-hlg-efb*
					+/+	VI	*sec-sei-cna-hla-hlg-efb*
Unknown (2654)	772/772, t345	V	1	+	+/+	VI	*sea-sei-cna-hla-hlg-efb*
Wound infection (284)	Singleton 4/2642, t064	V	1	–	+/+	IV	*hla-hlg-efb*
Wound infection (221)	30/30, t012	IV	3	+	+/+	VI	*sei-cna-hla-hlg-efb*
Wound infection (27/231)	30/503, t012	IV	3	+	+/+	VII	*sei-cna-hla-hlg*
Wound infection (296)	22/22, t005	IV	1	+	+/+	I	*sec-sei-cna-hla-hlg*
Wound infection (293)	22/1414, t1328	IV	1	+	+/+	I	*sei-cna-hla-hlg*
Wound infection (UC104)	22/22, Unk	IV	1	+	+/+	II	*sei-cna-hla-hlg-efb*
Wound infection (UC101)	22/22, t091				+/+		
Wound infection (UC463)	22/22, t309	IV	1	+	−/−	III	*sec-sei-cna-hla-hlg-efb*
Wound infection (2518)^∗^	121/120, t272		NT	+	+/+	VI	*sea-sei-cna-hla-hlg-efb*
**MSSA (*n* = 77)**							
Wound infection (2130)	772/772, t345		2	+	+/+	VI	*sec-cna-hla-hlg-efb*
Wound infection (2164)	772/772, t1839	UT^∗^	None	+	+/+	VI	*sea-sec-sei-cna-hla-hlg-efb*
Wound infection (2493)	772/1, t386		4	+	+/+	VI	*sei-cna-hla-hlg-efb*
Eye infection (N309852)	772/1, t098		3	–	+/+	VII	*sea-cna-hla-hlg*
Eye infection (518)	772/1, t693	UT^∗^	3	–	+/+	VII	*sea-sei-cna-hla-hlg-efb*
Eye infection (535,1636)	772/1, t127	UT^∗^	3	–	+/+	VII	*sea-sei-cna-hlg-efb*
					+/+	V	
Eye infection (831)	772/1, t127	UT^∗^	3	–	+/+	II	*sea-sei-cna-hla-hlg-efb*
Eye infection (1361)	772/1, t128	UT^∗^	3	–	+/+	VII	*sea-sec-sei-hla-hlg-efb*
Eye infection (1321)	772/1, t177	UT^∗^	3	–	+/+	VII	*sea-sei-cna-hla-hlg-efb*
Eye infection (1476)	772/1, t127		3	–	+/+	VIII	*sea-sei-cna-hla-hlg-efb*
Eye infection (1881)					+/+	I	
Eye infection (1503)	772/1, t127		3	–	+/+	VI	*sei-cna-hla-hlg-efb*
Eye infection (975)	772/1, t8078		3	–	+/+	VI	*sei-hla-hlg-efb*
Eye infection (1214)	772/772, t657		3	+	+/+	VI	*sea-sec-sei-cna-hla-hlg*
Healthy conjunctiva (N11OD)	772/1, t948	UT^∗^	None	–	+/+	I	*sea-sei-cna-hla-hlg-efb*
Healthy conjunctiva (N12OD)	772/1, t948		3	–	+/+	IV	*sea-cna-hla-hlg-efb*
Wound infection (2151)	30/714, t021		3	+	+/+	VI	*sei-cna-hla-hlg-efb*
Wound infection (2413)	30/1482, t386		3	+	+/+	IV	*sei-cna-hla-hlg-efb*
Eye infection (1196)	30/938, t021		3	+	+/+	IV	*sei-cna-hla-hlg-efb*
Eye infection (1850)					+/+	V	
Wound infection (2488)	121/121, t159		4	–	+/+	II	*sei-cna-hla-hlg-efb*
Eye infection (P832812)	121/121, t3204		4	+	+/+	V	*cna-hla-hlg*
Eye infection (P706434)	121/1964, t272		4	–	+/+	V	*sei-cna-hla-hlg*
Eye infection (917)	121/2160, t159		4	+	+/+	V	*cna-hla-hlg-efb*
Unknown (2657)	2884/2884, t4104		3	+	+/+	III	*hla-hlg-efb*
Eye infection (149)	2884/88, t5562		3	+	−/+	VI	*sei-hla-hlg-efb*
Eye infection (1764Y)	2884/88, t448		3	+	+/+	VIII	*sea-sei-hla-hlg-efb*
Eye infection (504, 1035, 1271)	5/5, t442		2	–	+/+	II	*sei-cna-hla-hlg-efb*
					+/+		*sei-hla-hlg-efb*
					+/+		*sei-hlg*
Eye infection (N303284)			None	–	+/+	I	*sei-cna-hla-hlg*
Eye infection (843)			2	–	+/+	VIII	*sei-hlg-efb*
Eye infection (1042)			2	–	+/+	VII	*sei-hla-hlg-efb*
Eye infection (1766, 1862)			1	–	+/+	VIII	*sei-hla-hlg*
					+/+		*sei-hla-hlg-efb*
Eye infection (1867)			1	–	+/+	VII	*sei-hla-hlg-efb*
Eye infection (1103)	5/5, t14912		2	–	+/+	V	*sei-hla-hlg-efb*
Eye infection (1306)	5/83, t442		2	–	+/+	II	*sei-hla-hlg-efb*
Eye infection (1424)	5/5, 8179		2	–	−/ +	VI	*sei-hla-hlg-efb*
Healthy conjunctiva (N9OD)	5/5, t010		2	–	+/+	VII	*sei-hla-hlg-efb*
Wound infection (17/201)	813/813, t10579		1	–	+/+	VII	*sei-cna-hla-hlg*
Wound infection (262)	813/291, t1149		1	–	+/+	VII	*hlg*
Eye infection (186)	22/22, t310		1	+	+/+	II	*sei-cna-hla-hlg-efb*
Healthy conjunctiva (N61OD)	22/22, t948	UT^∗^	1	+	+/+	VII	*sea-sei-hla-hlg-efb*
Eye infection (481)	Singleton 1/580, t14911		None	–	−/ +	V	*sei-cna-hla-hlg-efb*
Eye infection (N297214)	Singleton 2/45, t302		1	–	+/+	VII	*cna-hla-hlg*
Wound infection (2417)	Singleton 3/Unk, t021		None	–	−/−	VI	*sei-hla-hlg-efb*
Eye infection (1525,1545)	Singleton 5/72, t148		1	–	−/ +	VI	*sei-hla-hlg*
			None	–	−/ +	V	*sei-cna-hla-hlg-efb*
Wound infection (1/229, 861)	Singleton 6/789, t091		1	–	−/−	III	*sei-cna-hla-hlg*
			None	–	+/+	III	*sei-cna-hla-hlg-efb*
Wound infection (379)	Singleton 6/789, t2505		None	–	+/+	III	*sei-cna-hla-hlg-efb*
Eye infection (1603)	Singleton 6/789, t091		1	–	+/+	V	*sei-hla-hlg-efb*
Eye infection (1320)	Singleton 7/6, t657		1	–	+/+	III	*sei-cna-hla-hlg-efb*
Eye infection (1428)	Singleton 7/6, t4285		1	–	−/ +	VIII	*sea-sei-cna-hla-hlg-efb*
Eye infection (1698)	Singleton 7/6, t12406		1	–	+/+	VIII	*sea-sei-cna-hla-hlg-efb*
Healthy conjunctiva (N21OS)	Singleton 8/15, t084		2	–	+/+	IV	*sei-hla-efb*
Wound infection (2508)	Singleton 9/2885, t15579		4	+	+/+	III	*sei-cna-hla-hlg-efb*
Wound infection (2653)	Singleton 10/672, t3841		2	–	±	I	*sei-hla-hlg-efb*
Eye infection (N259615, N289378, 1049, 1506)	Singleton 10/672, t3841		1	–	+/+	I	*sei-cna-hla-hlg*
			None	–	+/+	I	*cna-hla-hlg*
			1	–	+/+	VII	*sei-hla-hlg-efb*
			1	–	+/+	VIII	*sei-hla-hlg-efb*
Eye infection (188, 1164, 1355, 1670)	Singleton 10/672, t1309		I	–	+/+	I	*sei-hla-hlg-efb*
			I	–	−/ +	II	*sei-hla-hlg-efb*
			I	–	+/+	I	*sei-cna-hla-hlg-efb*
			1	–	+/+	VIII	*sei-cna-hla-hlg*
Eye infection (884,1333)	Singleton 11/2233, t2663		3	+	+/+	VII	*sei-cna-hlg-efb*
					+/+		
Eye infection (1716OD, 1758)			3	+	+/+	IV	*sei-cna-hla-hlg*
					+/+		
Eye infection (1716OS, 1769)			3	+	−/+	VIII	*sei-cna-hla-hlg*
			4		+/+		
Eye infection (915, 1366, 1729)			3	+	+/+	VII	*sei-cna-hla-hlg-efb*
		UT^∗^	3	+	−/+	VII	*sei-hla-hlg*
			3	–	+/+	VIII	*sea-sei-cna-hla-hlg-efb*

*MRSA: methicillin-resistant *Staphylococcus aureus*; MSSA: methicillin-sensitive *Staphylococcus aureus*; CC: clonal complex; ST: sequence type; SSC: Staphylococcal cassette chromosome; agr: accessory gene regulator; Unk: unknown; UT: untypeable; NT: non-typeable; ^∗^Isolates with *ccr*AIB1 complex but lack *mec* complex; *pvl*: Panton-valentine leucocidin; *icaA*: intracellular adhesion gene A; *icaD*: intracellular adhesion gene D; *sea*: staphylococcal enterotoxin A; *sec*: staphylococcal enterotoxin C; *sei*: staphylococcal enterotoxin I; *cna*: collagen adhesion; *hlyA*: hemolysin A; *hlyG*: hemolysin G; *efb*: extracellular fibronectin binding protein.*

Ninety-one isolates comprising 26 MRSA and 65 MSSA were positive for both *icaA* and *icaD* genes, but five strains containing three MRSA and two MSSA were negative for both *icaA* and *icaD* genes. Two of the three MRSA isolates were positive for *icaA* gene, and another strain was positive for *icaD* gene. Similarly, nine of the 10 MSSA isolates were positive for *icaD* gene and one isolate for *icaA* gene, respectively (Table 4).

Also, a total of 25 toxin genes combinations was obtained with 109 strains belonging to 77 PFGE patterns, 32 sequence types (STs), 46 *spa*-types, and five *agr*-types. Twenty-three isolates belonging to five MRSA and 18 MSSA showed a toxin pattern comprising *sei-cna-hla-hlg-efb* genes. On the other hand, five MRSA and two MSSA showed another virulence pattern composed of *sea-sec-sei-cna-hla-hlg-efb* genes. The remaining isolates showed 23 different virulence gene patterns (Table 4).

### *Agr*-Typing

Of 109 *S. aureus* strains, 40 (36.7%) isolates belong to *agr*-I, 31 (28.4%) isolates to *agr*-III, 18 (16.5%) to *agr*-II, and seven (6.4%) belong to *agr*-IV; however, 13 (11.9%) isolates were not typeable by the method employed (Table 4). Of the 32 MRSA isolates, 20 (62.5%) belong to *agr*-I, five (15.6%) to *agr*-II, three (9.4%) to *agr*-III, and remaining isolates were untypeable. On the other hand, 28 of 77 (36.4%) MSSA isolates belong to *agr*-III, 20 (25.9%) to *agr*-I, 13 (16.9%) to *agr*-II, seven (9%) to *agr*-IV, and nine (11.7%) isolates were untypeable. There was a good correlation between virulence patterns and specific molecular types (Table 4). The *sea-sei-cna-hla-hlg-efb* was the dominant virulence pattern shown by MRSA belonged to SCC*mec* type UT6, and *agr* type I, followed by *sei-hla-hlg-efb* and *sei-cna-hla-hlg-efb* pattern showed by MSSA isolates belonged to *agr* type I and III, respectively (Supplementary Table S1).

### *Spa*-Typing

**FIGURE 1:**
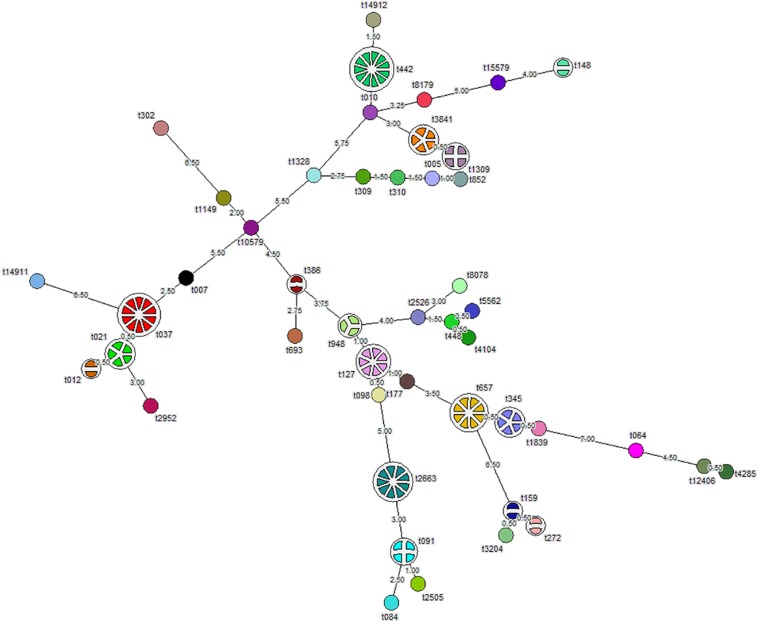
Minimum spanning tree (MST) showing 109 *S. aureus* isolates typed by *spa*-typing. Each node represents one *spa*-type, and the corresponding *spa*-type is given beside the node. The number of disks in a node indicates the number of isolates having a particular *spa*-type. The number provided on the string depicts the phylogenetic distance between two nodes. Length ≤ 1 is represented by dotted lines and more than one by solid lines.

Analysis of the aligned sequence of the polymorphic X region of *spa* gene using the *spa*-typing plug-in tool of Bionumerics 7 software showed 46 *spa*-types (Figure 1). MST analysis classified the strains into six major clusters, seven minor clusters, and 30 singletons. We designated cluster as a minor cluster that contained less than five but more than two strains. Of the 109 *S. aureus* isolates, 11 (10%) isolates belong to *spa*-type t442, 10 (9%) to t037, nine (8.2%) to t2663, eight (7.3%) to t657, six (5.5%) to t127, five (4.5%) isolates each to t345 and t3841, and four isolates each belong to t021, t091, and t1309. In addition, four (3.6%) isolates belong to t1309, three (2.7%) isolates belong to t948, and two (1.8%) each belong to t148, t386, t012, t159, and t272, respectively. Moreover, one isolate each of 30 strains belong to single *spa*-types, namely, t15579, t8179, t14912, t010, t852, t005, t310, t309, t1328, t302, t1149, t10579, t007, t14911, t2952, t693, t2526, t8078, t5562, t448, t4104, t177, t098, t084, t2505, t3204, t1839, t064, t12406, and t4285 (Figure 1). Whereas 10 of 32 (31.2%) MRSA isolates belong to t037, 11 of 77 (14.3%) MSSA isolates belong to t442. *S. aureus* strain ATCC 25923 showed *spa*-type t948 along with three test isolates. We found two novel *spa*-types, namely, t14911 and t14912 among *S. aureus* strains after submission of nucleotide sequences to the Ridom *spa* server. *Spa*-type t14912 showed a close association with major *spa*-type t442, but 14911 *spa*-type was diverse and unrelated. One of the isolates was not assigned any *spa*-type (Figure 1).

### Multi-Locus Sequence Typing (MLST)

**FIGURE 2:**
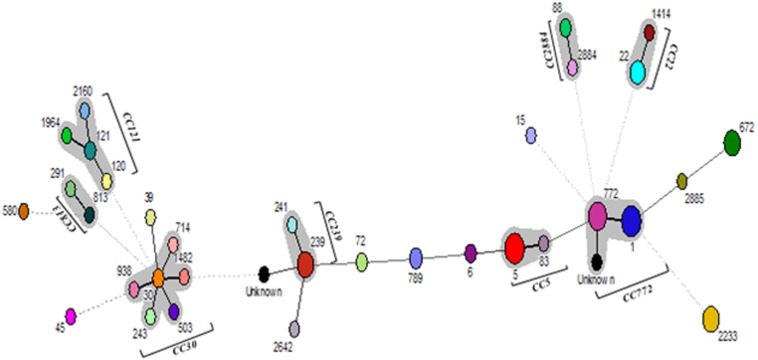
Minimum spanning tree (MST) showing the relationship between different STs assigned by the analysis of MLST data. Each node represents one sequence type, and the corresponding ST is given beside the node. The size of each node is directly proportional to the number of isolates included in that ST. Bold lines connect types that are identical for six loci, solid lines connecting types identical for ≥four but ≤six locus, and dotted lines connecting STs differing from each other by ≥four genes out of seven gene locus.

Multi-locus sequence typing of 109 *S. aureus* isolates showed 32 STs, eight CCs, and 12 singletons (Figure 2). The major ST comprised of ST1 (12.8%), ST5 (11.9%), ST772 (11%) followed by ST239 (9.2%), ST672 (8.3%), and ST2233 (8.3%). Also, we found two new allelic profiles designated as unknown not reported earlier among *S. aureus* strains (Supplementary Table S2). Of the eight CCs, CC5 contained 14 isolates, CC22 had eight isolates, CC30 had six isolates, CC121 had five isolates, CC239 had 11 isolates, CC772 had 26 isolates, CC813 had two isolates, and CC2884 contained four isolates, respectively. Of the major CCs, CC30 contained five STs, namely, ST30, ST503, ST714, ST938, and ST1482, CC121 contained four STs, namely, ST120, ST121, ST1964, ST2160, and CC772 had three STs, namely, ST772, ST1, and new unknown ST (Figure 2). Seven of the 32 (21.8%) of MRSA strains belong to ST239, *spa*-type t037, and SCC*mec* type UT6. However, 14 (18.2%) of MSSA strains possessing ST1 belong to different *spa*-types, namely, t127, t948, t177, t693, t098, and t386, of which few strains carry *ccr* complex but devoid of *mec* complex (Table 4). However, few isolates from eye infection and wound infection belong to CC239, ST239, and *spa*-type t037/t657. Reference strain of *S. aureus* ATCC25923 belonged to ST30 and CC30.

### Pulsed-Field Gel Electrophoresis

**FIGURE 3:**
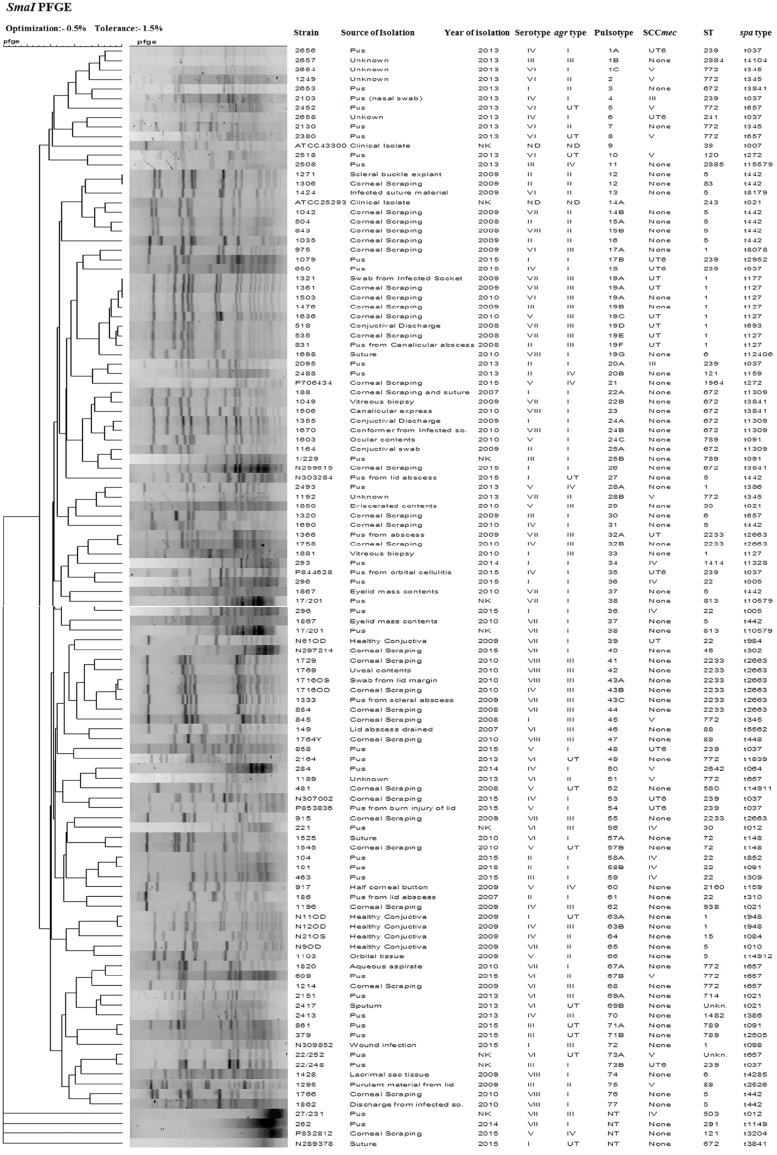
Dendrogram representation (Dice coefficient) for macro-restriction banding patterns of *S. aureus* strains isolated from different sources with ATCC reference strains, generated by pulsed-field gel electrophoresis of total chromosomal DNA digested with *Sma*I restriction enzyme and correlation between their pulsotype, ST, *spa*-type, SCC*mec* type, and *agr* type with information regarding their source and year of isolation.

*Sma*I-digested genomic DNA of *S. aureus* yielded bands classifying the 109 strains into 77 pulsotypes that includes two identical pairs (12 and 19A), three major clusters (1, 3, and 19), 17 minor clusters (14, 15, 17, 19, 20, 22, 24, 25, 28, 32, 57, 58, 63, 67, 69, 71, and 73), and 56 singletons. Four isolates were untypeable by the method employed. We found a total of 24 PFGE patterns among 32 MRSA isolates, of which one isolate was untypeable. Similarly, 77 MSSA isolates showed 53 PFGE patterns, of which three MSSA isolates were untypeable (Figure 3). MSSA isolates belonging to the major pulsotype 19 contained seven subtypes 19A, 19B, 19C, 19D, 19E, 19F, and 19G. These isolates were mostly from ocular infection and belong to ST1, *agr* type III, except one subtype 19G which belongs to ST6 and *agr* type I. *S. aureus* strain ATCC 25923 showed pulsotype 14. A dendrogram was generated using Bionumerics 7 software and percentage similarity with a cut-off of 80% and dice coefficients.

### Statistical Analysis

**FIGURE 4:**
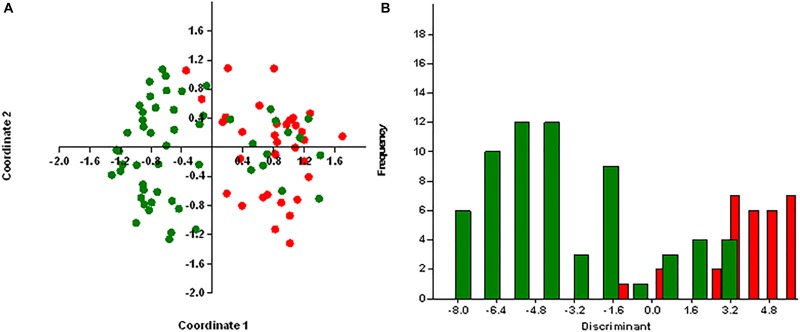
Results obtained by statistical analysis using antibiotic resistance genes. **(A)** Principle component analysis (PCoA) of methicillin-resistant *S. aureus* (MRSA, red) and methicillin-susceptible *S. aureus* (MSSA, green). **(B)** Discriminant analysis of MRSA (red) and MSSA (green). Negative values belong to MSSA isolates and positive values to MRSA isolates.

**FIGURE 5:**
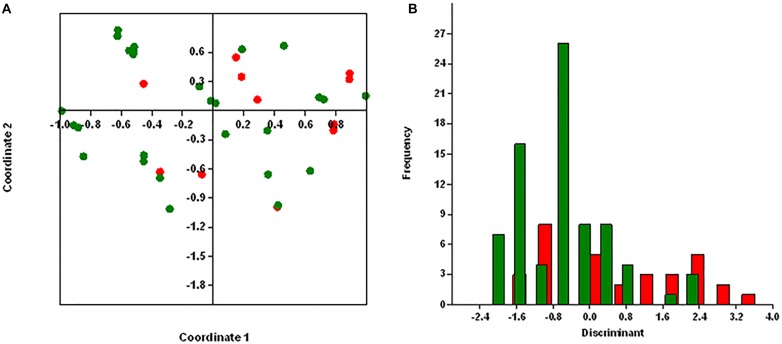
Results obtained by statistical analysis using virulence genes. **(A)** Principle component analysis (PCoA) of methicillin-resistant *S. aureus* (MRSA, red) and methicillin-susceptible *S. aureus* (MSSA, green). **(B)** Discriminant analysis of MRSA (red) and MSSA (green). Negative values belong to MSSA isolates and positive values to MRSA isolates.

Principal coordinates analysis segregates MRSA and MSSA isolates, except for few isolates with 25.75% of explained variance for antibiotic resistance genes (Figure 4A) and 26% for virulence genes (Figure 5A). We used axis one for the highest percentage of representation. DA graph showed that MRSA isolates grouped within more positive values, whereas MSSA isolates grouped within negative values for both antibiotic resistance genes and virulence genes (Figures 4B, 5B). Predominant biomarkers were determined by calculating the coefficient of discriminant function and considered when the value was equal to 0.5 or >0.5. For antibiotic resistance genes, MRSA isolates are discriminating in the biomarker of resistance to *ermA* (0.8407), *mphC* (2.0167), *tetK* (2.3495), *tetL* (2.0604), and *dfrA* (1.3116), whereas the MSSA isolates were discriminating in resistance to *aac(6’)/aph(2)* (−0.351), *aph3* (−2.7179), *ermC* (−0.8473), *tetM* (−0.522), *cat:pC221* (−2.421), *cat:pC223* (−6.601), *dfrB* (−0.443), and *dfrG* (−0.603). For virulence genes, MRSA isolates are discriminating in the biomarker of resistance to *icaA* (0.67169), *seA* (0.68593), *seC* (2.3245), *cnA* (0.90744), and *hlA* (0.54797). On the other hand, MSSA isolates were discriminating in resistance to *icaD* (−2.1945), *seI* (−0.58795), and *hlG* (−1.4999). PCoA and discriminant function of antibiotic resistance and virulence genes of *S. aureus* isolates with source and place of isolation was heterologous and complex (data not shown).

## Discussion

We used hexaplex PCR for detection of MRSA and MSSA isolates along with the presence of *mecA*, *pvl*, *czrC*, and *qacA/B* genes. We found a good correlation between oxacillin resistance and the presence of the *mecA* gene. However, one isolate showing resistance to oxacillin and lack *mecA* gene indicate the occurrence of different mechanism of methicillin resistance. Twenty of 31 MRSA and 23 of 77 MSSA isolates were positive for *pvl* gene indicating the prevalence of *pvl* gene among MRSA strains from the wound and eye infections. This finding is in contrast to those who did not find such correlation among clinical isolates (Shittu et al., 2011); therefore, it cannot be used as a reliable marker for MRSA. The presence of *czrC* and *qacA/B* genes among the number of MRSA isolates indicates their possible association with the *mecA* gene; however, further investigation is required to authenticate these findings.

Coagulase gene typing has been used to characterize *S. aureus* strains. Hwang and Kim (2007) showed the presence of coagulase serotype II among 54.4% MRSA and serotype VII among 30.9% MSSA. In contrast, we found serotype VII was present among 22% of MSSA isolates and serotype VI in 28.1% of MRSA isolates. These observations thus suggest that there is a difference in the presence of serotypes with regard to MRSA and MSSA.

Genetic determinants study among *S. aureus* showed a good correlation between resistance to aminoglycosides, chloramphenicol, clindamycin, erythromycin, trimethoprim, and tetracycline, and the presence of corresponding resistance genes. In this study, we found 85.3% strains showing resistance to chloramphenicol carried the *pC221* gene; however, some of these strains also carried either *pC223* or *pC194* or both genes. Although one of 109 strains sensitive to chloramphenicol did not carry any of these genes, 13.8% strains showing sensitivity to chloramphenicol carried either *pC221* or *pC223* genes. These observations thus suggest that chloramphenicol sensitive strains carrying antibiotic resistance genes can develop resistance against this drug on exposure.

The aminoglycoside-modifying enzyme, encoded by *aac (6’)-aph(2”)* gene, is responsible for resistance against aminoglycosides (Vanhoof et al., 1994). Besides, two other genes encoding for *aph(3.III)* and *ant(4, IV)* are accountable for aminoglycoside resistance, but their frequency is less compared to *aac(6’)-aph(2”)* among staphylococci (Busch-SØRensen et al., 1996). In this study, we found 41.3% *S. aureus* possesses both *aac(6’)-aph(2”)* and *aph (3, III)* genes and 8.3% contained *aph (3, III)* gene and showed phenotypic resistance to gentamycin. These findings thus suggest that there are strains which harbor aminoglycoside resistance genes other than *aac(6’)-aph(2”)* and few strains had *aph (3, III)* only. At least 47.7% strains of *S. aureus* that were sensitive to aminoglycosides contain either *aph (3, III)* or aac*(6’)-aph(2”)* or both; however, three strains susceptible to gentamycin lack resistance genes. These findings are in contrast to those workers who reported that all aminoglycoside-resistant strains carried *aac(6’)-aph(2”)* (Price et al., 1981; Lovering et al., 1988; Dornbusch et al., 1990; Vanhoof et al., 1994; Martineau et al., 2000). The presence of aminoglycoside resistance gene among gentamycin sensitive isolates of *S. aureus* indicates that there is likely hood development of aminoglycoside resistance among *S. aureus* upon exposure to these drugs.

Similarly, 83.4% strains of *S. aureus* resistant to erythromycin harbored any of the four genes, namely, *erm*A, *ermB*, *ermC*, and *msrA*; however, an strain sensitive to erythromycin did not carry any of the genes. Previously, it was reported that the *ermA* gene is dominant among erythromycin resistance genes in *S. aureus* (Kaur and Chate, 2015). In contrast, we found the presence of the *ermC* gene in 83.4% strains compared to 49% of *ermA* gene. Kaur and Chate (2015) reported that majority of MRSA strains showed constitutive MLSB (cMLSB) resistance; however, two isolates had inducible MLSB (iMLSB) phenotype. In this study, 64.5% MRSA and 37.1% MSSA strains belong to iMLSB phenotype; however, 35.4% of MRSA and 43.5% of MSSA strains belong to cMLSB phenotype. This difference could be due to less number of MRSA isolates used in the study, and MSSA isolates were multidrug resistant. Seventeen strains showing sensitivity to erythromycin harbored one of the resistance genes, and one of the strains resistant to erythromycin did not possess any of the resistance genes to indicate that these strains are likely to develop resistance and mediated by an unknown mechanism.

About trimethoprim resistance, 67.8% strains harbored any of the three genes, namely, *dfrA*, *dfrB*, and *dfrG*. The remaining strains showing sensitivity to trimethoprim also carried all or one of the three genes. In this study, 73 of 74 trimethoprim resistance strains possess *dfrG* and *dfrB* genes; 45 strains carried the *dfrA* gene. These findings are in contrast to those who reported the presence of the *dfrG* gene in 92% strains, *dfrA* in 7% strains, and one strain carried a *dfrB* among trimethoprim resistance strains in a travel-associated skin and soft tissue infection study in Europe (Nurjadi et al., 2015).

Like other antibiotic resistance, 26.6% phenotypic resistance strains carried one or all the three tetracycline resistance genes, namely, *tetK*, *tetL*, and *tetM*. One of the strains sensitive to tetracycline was devoid of carrying any genes. However, the majority (69.7%) strain showing sensitivity to tetracycline carried one or all three resistance genes indicating that these isolates could develop resistance after exposure to an antibiotic. From this study, it is clear that erythromycin and gentamicin were least active; however, vancomycin and clindamycin were the most effective drugs. These results corroborate the finding of Pai et al. (2010), who also reported that vancomycin and clindamycin are the most effective drugs.

SCC*mec* type V was predominant type among MRSA strains followed by SCC*mec* type UT6, IV, and III, respectively. This finding is similar to Nadig et al. (2012), who also reported the prevalence of SCC*mec* type V among isolates from eye infections. To our knowledge, we are the first to inform of the presence of SCC*mec* type UT6 among *S. aureus* from India. The combination of SCC*mec* IV, V, and *pvl* gene was reported as the genetic markers for a community-associated MRSA (Bhutia et al., 2015). Similarly, our study showed the presence of SCC*mec* V (40.6%), IV (21.9%), and *pvl* (64.5%); therefore it can be used as a marker for hospital-associated infections. However, new UT6 SCC*mec* type is emerging in India. Many untypeable strains carried *ccr* complex but no *mec* complex. This observation thus suggests the ability of such strains to acquire *me*c complex and became a known or unknown SCC*mec* type.

A total of 25 unique toxin combination was found among *S. aureus* strains, of which at least one toxin gene was present in a given strain. Sotto et al. (2008) reported the presence of *sei* and *sea* genes in *S. aureus* isolated from diabetic foot ulcer. Similarly, we found the presence of *sei* and *sea* genes in both MRSA and MSSA strains. Although we noted the high percentage of *hlg* (98%) and *hla* (92.6%) among in *S. aureus* comprising both MSSA and MRSA, other workers reported the presence of these genes in mupirocin resistant in MRSA isolates in China (Liu et al., 2012). Moreover, the distribution of virulence genes with regards to source and place of isolation was complex. Gowrishankar et al. (2016) reported the isolation of 84% MRSA strains carrying *icaADBC* genes from patients with pharyngitis. Also, in this study, 81.3% MRSA and 84.4% MSSA carrying *icaA/icaD* genes were isolated from the eye and wound infections (Supplementary Table S3). Absence of *icaA/icaD* genes in *S. aureus* strains was similar to those of the previous report (Agarwal and Jain, 2013).

Several molecular genotyping tools are used to trace the origin of the strain, and distribution of CC with regard to methicillin-resistant, methicillin-sensitive, sources and place of isolation. We determined the population structure of *S. aureus* isolated from ocular and wound infections from different parts of India using MLST, *agr*-typing, *spa*-typing, and PFGE.

Multi-locus sequence typing analysis showed the presence of six major ST(s) comprising ST1, ST5, ST772, ST239, ST672, and ST2233, respectively. While ST239-MRSA-UT6 was the typical type among MRSA isolates from wound infection, ST772-MRSA-V were from eye infections (Nadig et al., 2012). Similarly, ST772-SCC*mec*-V were reported slowly replacing multidrug resistant ST239-SCC*mec*-III in Asian studies (D’Souza et al., 2010). This finding is in contrast to Suzuki et al. (2012), who reported the presence of ST5 and ST764 among MRSA strains from the infected eye and healthy conjunctiva sacs. Also, Mohammadi et al. (2014) showed emergence of SCC*mec*-III with variable antimicrobial resistance profiles in Iran. We found ST772-MRSA-V with *spa*-type t345 and t657 belonging to dominant CC772 among wound infection isolates. Besides, we reported two new *spa*-types among *S. aureus* strains from India.

There were eight CCs, namely, CC30, CC121, CC772, CC813, CC239, CC28841, CC22, and CC5 present among *S. aureus* represents different PFGE clusters. CC30 and CC121 comprising different STs were almost equally distributed among MRSA and MSSA isolates. Whereas CC772-ST772 was dominant among MRSA, CC772-ST1 was prevalent among MSSA isolates. Similarly, CC239-ST239 and CC22-ST22 were prevalent among MRSA isolates and CC5-ST5, CC813-ST813, and CC28841-ST28841 were more commonly found in MSSA isolates. The prevalent CC among Varanasi isolates (mostly wound infection) were CC772 followed by CC239 besides the presence of CC30, and CC121. However, isolates from wound infection from Delhi showed varied results. Whereas AIIMS isolates showed CC CC30, CC22, and CC813, UCMS isolates showed the presence of CC239 and CC22 CCs. Interestingly isolates from Hyderabad (eye infection) had CC239 but isolates from Bhubaneswar (eye infection) showed the presence of CC772, CC5, CC2884, and CC30.

Mobasherizadeh et al. (2019) reported the prevalence of CC5 and CC30 and other CCs among MRSA isolates isolated from nasal carriage in Iranian hospitals. Similarly, CC8, CC121, CC1, CC45, and CC5 were reported in MRSA isolates from Malaysia (Ghasemzadeh-Moghaddam et al., 2011). These observations indicate the existence of different CCs in India and Asian countries. MLST and *spa*-typing was better than PFGE and toxin genotyping a finding unusual from those who reported a good correlation between various typing schemes. Overall, there was diversity in genotypes, antimicrobial resistance, and virulence determinants among MRSA and MSSA strains.

From this study, it is clear that *S. aureus* strains sensitive to antibiotics but carried antibiotic resistance genes could develop resistance upon exposure to antibiotic(s), and vancomycin and clindamycin were the most effective drugs. ST239-SCC*mec* UT6/t035 were dominant clones among *S. aureus*. There was diversity in genotypes, antimicrobial resistance, and virulence determinants among MRSA and MSSA strains, therefore suggests continuous surveillance of multidrug-resistant strains circulating in the community/hospitals in India, to take adequate measures to control the infection.

## Data Availability Statement

The raw data supporting the conclusions of this article will be made available by the authors, without undue reservation, to any qualified researcher.

## Ethics Statement

The studies involving human participants were reviewed and approved by the Institutional Review Board (IRB) of LV Prasad Eye Institute (LEC/08/110/2009) and by the Institute Ethics Sub-Committee (IESC) of All India Institute of Medical Sciences, New Delhi (IESC/T-34/2013), and the data were analyzed anonymously and reported. The patients/participants provided their written informed consent to participate in this study.

## Author Contributions

SA, SJ, SS, and DS conceived the experiments. SA, SJ, and SP conducted the experiments. SA, SJ, SP, SS, BD, GN, NS, and DS analyzed the results. KN performed statistical analysis. SA, SJ, and DS wrote the manuscript. All authors reviewed and approved the manuscript.

## Conflict of Interest

The authors declare that the research was conducted in the absence of any commercial or financial relationships that could be construed as a potential conflict of interest.
