# Supplementary material for: Antibiotic Susceptibility, Virulence Pattern, and Typing of Staphylococcus aureus Strains Isolated From Variety of Infections in India
Source: Front Microbiol. 2019 Dec 4;10:2763. doi: 10.3389/fmicb.2019.02763 (PMC6904308; doi:10.3389/fmicb.2019.02763)
Supplement: Supplementary file 1 [file Data_Sheet_1.pdf]

SUPPLEMENTAL MATERIAL

TABLE S1. Virulence gene profiles and *agr* types among methicillin-resistant and –sensitive isolatesof *Staphylococcus aureus*.

agr type	Toxin gene profile	No. (%) of MRSA	No. (%) of MSSA
I	*sei-cna-hla-hlg-efb*	3 (9.6%)	3 (3.8%)
	sea-sei-cna-hla-hlg-efb	4 (12.9%)	1 (1.2%)
	sei-cna-hlg-efb	1 (3.2%)	0
	cna-hla-hlg	0	1 (1.2%)
	hla-hlg-efb	1 (3.2%)	0
	sec-sei-cna-hla-hlg	1 (3.2%)	0
	hlg	0	1 (1.2%)
	sea-cna-hla	1 (3.2%)	0
	sea-cna-hla-hlg-efb	2 (6.4%)	0
	sea-hla-hlg-efb	1 (3.2%)	0
	sec-sei-cna-hla-hlg-efb	1 (3.2%)	0
	sei-hla-hlg-efb	1 (3.2%)	7 (9%)
	sei-hla-hlg	0	2 (2.5%)
	sei-cna-hla-hlg	3 (9.6%)	4 (5.1%)
	sea-sec-sei-cna-hla-hlg-efb	1 (3.2%)	0
	sea-sei-hla-hlg-efb	0	1 (1.2%)
II	*sec-cna-hla-hlg-efb*	0	1 (1.2%)
	sec-sei-cna-hla-hlg-efb	2 (6.4%)	0
	sea-sei-cna-hla-hlg-efb	1 (3.2%)	1 (1.2%)
	sea-sec-sei-cna-hla-hlg-efb	1 (3.2%)	0
	sei-cna-hla-hlg-efb	0	1 (1.2%)
	sei-hla-efb	0	1 (1.2%)
	sei-hlg-efb	0	1 (1.2%)
	sei-hlg	0	1 (1.2%)
	sei-hla-hlg-efb	1 (3.2%)	7 (9%)
III	*sei-cna-hla-hlg-efb*	1 (3.2%)	6 (7.7%)
	hla-hlg-efb	0	1 (1.2%)
	sea-cna-hla-hlg	0	1 (1.2%)
	sea-sei-cna-hla-hlg-efb	0	6 (7.7%)

	sea-sei-cna-hlg-efb	0	2 (1.2%)
	sea-sec-sei-cna-hla-hlg-efb	1 (3.2%)	0
	sei-hla-hlg-efb	0	2 (2.5%)
	sea-sec-sei-cna-hla-hlg	0	1 (1.2%)
	sea-sec-sei-hla-hlg-efb	0	1 (1.2%)
	sea-sei-cna-hla-hlg	0	0
	sei-cna-hla-hlg	1 (3.2%)	3 (3.8%)
	sei-cna-hlg-efb	0	2 (2.5%)
	sei-hla-hlg	0	1 (1.2%)
	sea-sei-hla-hlg-efb	0	1 (1.2%)
	sea-cna-hla-hlg-efb	0	1 (1.2%)
IV	*sei-cna-hla-hlg-efb*	0	3 (3.8%)
	cna-hla-hlg	0	1 (1.2%)
	sei-cna-hla-hlg	0	2 (2.5%)
	cna-hla-hlg-efb	0	1 (1.2%)
NT	*sea-sec-sei-cna-hla-hlg-efb*	3 (9.6%)	1 (1.2%)
	sei-hla-hlg-efb	0	1 (1.2%)
	sea-sei-cna-hla-hlg-efb	1 (3.2%)	1 (1.2%)
	sei-cna-hla-hlg	0	1 (1.2%)
	cna-hla-hlg	0	1 (1.2%)
	sei-cna-hla-hlg-efb	0	4 (5.1%)

NT: Non-typable

TABLE S2. Clonal Complex, Sequence types and spa-types of methicillin-resistant and methicillin-sensitive *Staphylococcus aureus* isolates.

Clonal Complex	ST	*spa* type	No. (%) of MRSA	No. (%) of MSSA
CC30	ST30	t012	1 (3.2%)	0
	ST503	t012	1 (3.2%)	0
	ST714	t021	0	1 (1.2%)
	ST938	t021	0	2 (2.5%)
	ST1482	t386	0	1 (1.2%)
CC121	ST121	t3204	0	1 (1.2%)
		t159	0	1 (1.2%)
	ST1964	t272	0	1 (1.2%)
	ST120	t272	1 (3.2%)	0
	ST2160	t159	0	1 (1.2%)
CC772	ST772	t657	5 (16.1%)	1 (1.2%)
		t1839	0	1 (1.2%)
		t345	3 (9.6%)	1 (1.2%)
	ST1	t177	0	1 (1.2%)
		t386	0	1 (1.2%)
		t098	0	1 (1.2%)
		t127	0	6 (7.7%)
		t693	0	1 (1.2%)
		t128	0	1(1.2%)
		t8078	0	1 (1.2%)
		t948	0	2 (2.5%)
	Unknown	t657	1 (3.2%)	0
CC813	ST813	t10579	0	1 (1.2%)
	ST291	t1149	0	1 (1.2%)
CC239	ST239	t037	9 (29%)	0
		t2952	1 (3.2%)	0
	ST241	t037	1 (3.2%)	0
CC2884	ST2884	t4104	0	1 (1.2%)
	ST88	t448	0	1 (1.2%)
		t5562	0	1 (1.2%)
		t2526	1 (3.2%)	0
CC22	ST22	t005	1 (3.2%)	0

		t845	1 (3.2%)	0
		t091	1 (3.2%)	0
		Unknown	1 (3.2%)	0
		t309	1 (3.2%)	0
		t310	0	1 (1.2%)
		t948	0	1 (1.2%)
	ST1414	t1328	1 (3.2%)	0
CC5	ST5	t442	1 (3.2%)	9 (11.6%)
		t14912	0	1 (1.2%)
		t8179	0	1 (1.2%)
		t010	0	1 (1.2%)
	ST83	t442	0	1 (1.2%)
Singleton 1	ST580	t14911	0	1 (1.2%)
Singleton 2	ST45	t302	0	1 (1.2%)
Singleton 3	Unknown	t021	0	1 (1.2%)
Singleton 4	ST2642	t064	1 (3.2%)	0
Singleton 5	ST72	t148	0	2 (2.5%)
Singleton 6	ST789	t091	0	2 (2.5%)
		t2505	0	2 (2.5%)
Singleton 7	ST6	t657	0	1 (1.2%)
		t4285	0	1 (1.2%)
		t12406	0	1 (1.2%)
Singleton 8	ST15	t084	0	1 (1.2%)
Singleton 9	ST2885	t15579	0	1 (1.2%)
Singleton 10	ST672	t3841	0	5 (6.4%)
		t1309	0	4 (5.1%)
Singleton 11	ST2233	t2663	0	9 (11.6%)

CC: Clonal complex; ST: Sequence type; MRSA: Methicillin-resistant *Staphylococcus aureus*; MSSA: Methicillin-sensitive *Staphylococcus aureus.*

TABLE S3. Distribution of *ica* genes in relation to *agr* types, SCC*mec* types and MLST of *Staphylococcus aureus* strains.

Genetic characteristics	No. (%) of isolates positive for genes encoding for	
***agr* group**	*icaA*	*icaD*
Agr I	35	36
Agr II	17	16
Agr III	28	32
Agr IV	6	6
NT	8	10
**SCC*mec* type**		
III	2	2
IV	6	6
V	13	14
UT6	8	6
**MLST**		
ST772	11	12
ST239	9	7
ST88	2	3
ST5	12	13
ST22	5	5
ST1482	1	1
ST2233	7	9
ST1	14	14
ST120	0	0
ST121	2	2
ST672	8	8
ST789	3	3
ST813	1	1
ST291	1	1
ST72	0	2
ST2885	1	1
ST2884	1	1
ST45	1	1

ST243	1	1
NK	0 (2)	0 (2)
ST2642	1	1
ST83	1	1
ST580	0	1
ST714	1	1
ST938	2	2
ST15	1	1
ST1964	1	1
ST2160	1	1
ST503	1	1
ST1414	1	1
ST6	2	3
ST241	1	Nil

agr: accessory gene regulator; SCC: staphylococcal cassette chromosome; MLST: Multi-locus sequence typing; ST: Sequence type; NT: not typeable; UT: untypeable; NK: Unknown
